# Statistical Method for Dental Clinics for Determining Presence and Stage of Periodontitis with aMMP-8 Mouth Rinse Point-of-Care Test and Digital Reader

**DOI:** 10.3390/dj13110508

**Published:** 2025-11-03

**Authors:** Miika Penttala, Ismo T. Räisänen, Dimitra Sakellari, Andreas Grigoriadis, Timo Sorsa

**Affiliations:** 1 Department of Oral and Maxillofacial Diseases, Head and Neck Center, University of Helsinki and Helsinki University Hospital, 00290 Helsinki, Finland; ismo.raisanen@helsinki.fi (I.T.R.); timo.sorsa@helsinki.fi (T.S.); 2 Department of Preventive Dentistry, Periodontology and Implant Biology, Dental School, Faculty of Health Sciences, Aristotle University of Thessaloniki, 541 24 Thessaloniki, Greece; dimisak@dent.auth.gr (D.S.); andreasgrigor@gmail.com (A.G.); 3 Dental Sector, 424 General Military Training Hospital, 564 29 Thessaloniki, Greece; 4 Division of Oral Diseases, Department of Dental Medicine, Karolinska Institutet, 171 77 Stockholm, Sweden

**Keywords:** periodontitis, staging, aMMP-8, POCT, logistic regression

## Abstract

**Background/Objectives:** This study proposes a framework for building a statistical prediction model for dental clinics to facilitate the diagnosis of periodontitis and its stages. The method is based on active-matrix metalloproteinase-8 (aMMP-8) mouth rinse point-of-care testing (POCT). **Methods:** A complete model was created within a three-step modeling scenario: (i) the first function differentiates healthy patients from those with periodontitis; (ii) the second function differentiates stage I and II patients from stage III patients; and (iii) the third function separates stage I and II patients from each other. The model was developed using logistic regression analysis, and the aMMP-8 POCT results utilized in the predictive functions were obtained from an Oralyzer digital reader. Sample data comprised 149 adult patients who visited dental clinics in Thessaloniki, Greece. **Results:** Patients without periodontitis were identified in 74.2% of cases (95% CI: 55.1–87.5%). Patients with periodontitis were revealed with a success rate of 94.1% (95% CI: 87.7–97.4%), and of these, the correct stage was determined in 71.2% of cases (95% CI: 61.7–79.2%). The complete model was tested on the same patient data from which it was formed. **Conclusions:** The results of the study showed that logistic regression can be used in the development of a model for dental clinics to reveal and stage periodontitis with sufficient accuracy. In the complete model created, aMMP-8 mouth rinse POCT results in ng/mL, visible plaque index (VPI), and the information on the patient’s missing teeth were statistically important factors in determining the presence and stage of periodontitis.

## 1. Introduction

Periodontitis is a very common and highly prevalent but largely hidden disease; it is estimated that one in two dentate adults worldwide suffers from some category of periodontitis [1,2]. The slowly progressing infection-induced inflammation condition leads to the destruction of tissue and the resorption of alveolar bone [1,3]. An interesting aspect of periodontitis is that it is associated with several other diseases, such as diabetes and cardiovascular disease [1,3,4,5,6].

This article presents a method that offers statistical advantages, particularly for dental professionals, in determining and staging periodontitis. The predictive factors of the model can be directly utilized from the clinic’s computer system, and the method is intended for dental professionals, such as dentists, dental hygienists, and dental nurses.

The method is entirely statistical and includes three novel polynomial functions that form a predictive model. Its contribution to supporting the work of dental professionals is to provide statistical results to improve the diagnosis of periodontitis, as problems have been reported in diagnosing all stages of the disease. It is noteworthy that, for example, errors in clinical attachment level measurement make it particularly difficult to classify early periodontitis [7]. Diagnoses have been found to be more accurate in detecting advanced periodontitis (stages III and IV) compared to patients with stages I and II, but separating stages III and IV can still be difficult [8]. In general, scientists have directly encouraged the development of software solutions so that decision-making algorithms could be used to ensure the correct diagnosis of periodontitis [8,9,10]. In order to improve diagnostic agreement with standardized criteria, examining ‘gray areas,’ and education are an essential part of the proposed solutions [8,11,12].

In the approach of this study, active-matrix metalloproteinase-8 (aMMP-8) mouth rinse point-of-care testing (POCT) is at the core of the modeling. It has been shown already from the 1990s that aMMP-8, found in oral fluids, functions as a biomarker for ongoing tooth-supporting tissue destruction [13,14,15,16]. To investigate periodontitis, we used a sample of 149 adult patients attending dental clinics in Thessaloniki, Greece [17,18].

Predictive models related to the model presented in this study have been previously published [19,20]. This study utilizes continuous mouth rinse aMMP-8 POCT concentration results obtained with the Oralyzer digital reader as predictive factors for the functions. This represents a fundamental difference compared to earlier published rapid home-use models that were designed to use a single aMMP-8 mouth rinse cut-off 20 ng/mL test result [21]. The model in this study is especially intended for dental clinics. Also, explanatory factors such as bleeding on probing (BOP) or weight-adjusted waist index (WWI), which have not been used as variables in previous models, were examined as part of different combinations of predictors to achieve the most accurate prediction results for periodontitis and its stages.

Furthermore, the patient segmentation process developed in this study is completely novel and allows for the differentiation of stage I patients from stage II patients in particular. By expanding the research horizon to specifically include the development of a predictive function for stage I periodontitis, the aim of the study is to provide dental researchers with new ideas for detecting patients with early-stage periodontitis (i.e., stage I). Starting dental treatment as early as possible is important, as disease progression can be mitigated or halted by, for example, scaling and root planning (SRP) [22,23,24,25,26,27].

The primary objective of this study is to improve the accuracy of statistical prediction models for periodontitis and its stages by utilizing specific data provided by dental clinics. Furthermore, the study’s predictive statistical model can help optimize dental care resources by improving timely treatment of patients at different stages. The model aims to provide dental professionals with all possible statistical benefits in their work.

## 2. Materials and Methods

### 2.1. Hypothesis and Modeling

**Figure 1:**
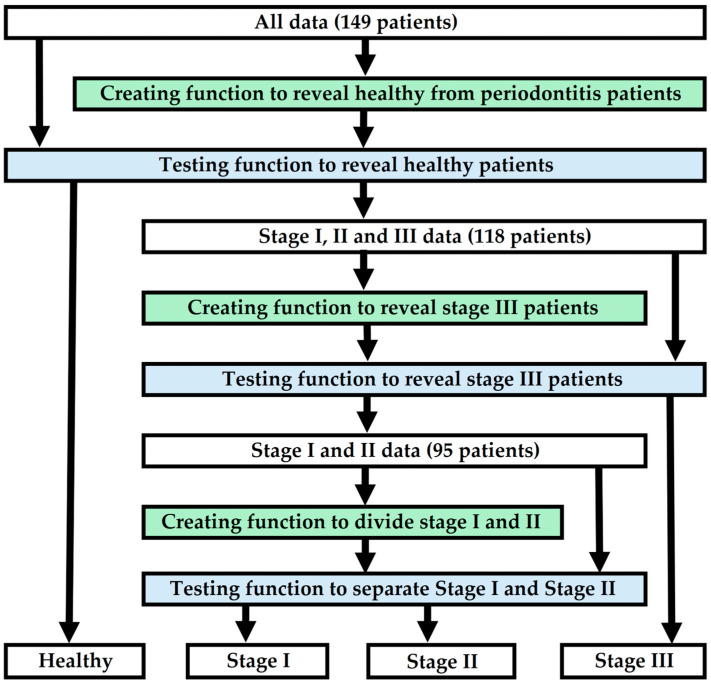
A flowchart depicting the modeling of the research.

The hypothesis of the study is that by using five to twenty predictive factors available from dental clinics, a model can be created to determine periodontitis and its stages at a sufficient level. The aim of the study is to present a detailed framework for building a statistical predictive model, specifically for dental professionals, to facilitate the diagnosis of periodontitis and its stages. The method involves creating polynomial functions using logistic regression analysis. The study design first distinguishes healthy patients from periodontitis patients (stages I, II and III). After this, the model reveals stage III patients. Finally, stage I and II patients are distinguished from each other. The predictive functions of the model must achieve sufficiently acceptable performance, which is examined, for example, through sensitivity, specificity, or chi-square goodness-of-fit tests, after which they form a complete model that can predict periodontitis and its stage. Figure 1 illustrates a diagram showing how to create and test a model that reveals periodontitis and its stage.

### 2.2. aMMP-8 Point-of-Care Testing (POCT)

The aMMP-8 POCT can measure progressive collagenolytic periodontal and peri-implant attachment loss caused by aMMP-8 in five minutes without obtaining an invasive tissue sample or the risk associated with bacteremia [14,15,16,17,20,28,29,30,31,32,33,34,35,36,37,38,39]. The predictive functions of the model in this study utilize aMMP-8 POCT results obtained from the Oralyzer digital reader. The ability of mouth rinse aMMP-8 concentrations to discriminate between different patient sample data was calculated using the Matthews correlation coefficient (MCC) for binary data and cut-off values of 10, 15, 20, 25, 30, 35, 40, 45 and 50 ng/mL were investigated [40]. The aMMP-8 cut-off value for each patient data discrimination scenario was selected based on how it best fit the other predictors while providing the most accurate result. The study also examined the possibility of using aMMP-8 concentration as a continuous value for the prediction of periodontitis and its stages. aMMP-8 mouth rinse test results were examined as a continuous value at 5-point intervals in ng/mL.

Lateral-flow chairside/PoCPerioSafe^®^ aMMP-8 mouth rinse point-of-care tests (POCT) were discovered in Finland and further developed in Germany. The digital reader (ORALyzer^®^) and the chairside/PoCPerioSafe^®^ test are manufactured by Medix Biochemica Ltd., Espoo, Finland, and Dentognostics GmbH, Solingen, Germany. The test method is based on monoclonal antibody technology and the aMMP-8 POCT has been independently and repeatedly confirmed by many parties [14,15,16,28,30,31,32]. These tests are similar in principle to traditional pregnancy and HIV tests and have been successfully clinically validated in Chile, Finland, Germany, Holland, India, Italy, Mali, Nigeria, Sweden, Türkiye and the United States [28,29,38,39,41,42,43].

The test consists of a series of steps. Before sampling, a 30 s pre-rinse with potable water, followed by a 60 s waiting period. Next, 5 mL of test solution (oral fluid sample) is collected after 30 s of rinsing. Three drops of this sample are transferred to the test system, which is placed in a digital reader. The reader provides the aMMP-8 concentration (ng/mL) within five minutes [17,19,20]. In this present study, aMMP-8 POCT was performed on patients by an experienced and trained researcher [AG].

### 2.3. Other Oral Health Parameters

In this study, the amount of dental plaque on the tooth surface was used to predict periodontitis and its stages. Plaque was evaluated as an independent variable, either in isolation or together with other factors such as the number of teeth. Visible plaque index (VPI) was applied in the procedure [44]. It was calculated by adding together the surfaces with detected dental plaque, and this sum was then divided by the total number of surfaces. The initiation of periodontitis can be described as the accumulation and microbial dysbiosis of dental plaque biofilm [3]. It is worth noting that the study sample did not include information on microbial specificity, and this issue could not be investigated in the creation of prediction functions [45].

The study also investigated bleeding on probing (BOP) and its relationship to periodontitis, especially its stages. This experimental design was performed even though BOP score is not considered an indicator of the severity of inflammation [46]. BOP was considered as a candidate variable, which could be continuous, binary, or part of a composite variable with other predictors. BOP is part of the new periodontitis classification system, although it should be noted that it is not used in the actual classification of periodontitis [7,47,48]. The periodontal tissues were examined by assessing BOP to the bottom of the clinical pocket or sulcus using a periodontal automated probe (Florida Probe, Florida Probe Corporation, Gainesville, FL, USA). The BOP score was calculated by dividing the number of bleeding gingival units by the total number of examined sites for each patient [49].

As mentioned earlier, the number of teeth in a patient was studied as a predictor of periodontitis, for example, together with dental plaque. Periodontitis is the leading cause of tooth loss among adults across the world, and the extent of tooth loss due to periodontitis is used in staging of the disease [7,47,48]. Therefore, the study investigated how the patient’s tooth count would affect the model’s results. A patient’s dentition was considered complete if the number of teeth was counted as 28 (excluding wisdom teeth).

### 2.4. Periodontitis Classification

All patients included in this study were classified based on the 2018 classification of periodontal diseases [7,47,48]. In the separation of the sample data, the status of different periodontitis patients was treated as a binary variable. When healthy patients were to be differentiated into their own group, patients suffering from periodontitis were marked with the binary value one, while patients with no evidence of periodontitis were marked with the value zero. The same binary principle was also applied to distinguish, for example, stage I and stage II patients from each other.

Staging of periodontitis can be summarized following the severity and complexity below [7,12]:

Stage I: The diagnosis requires a Clinical Attachment Loss (CAL) of 1–2 mm, less than 15% Radiographic Bone Loss (RBL) in the coronal third of the root, and no tooth loss due to periodontitis. Periodontal Probing Depth (PPD) of up to 4 mm and bone loss mostly horizontal.

Stage II: CAL 3–4 mm, RBL of 15–33% in the coronal third of the root, and no tooth loss related to periodontitis. PPD up to 5 mm and bone loss mostly horizontal.

Stage III: CAL 5 mm or greater, RBL reaches the mid-third or beyond, and up to four teeth have been lost due to periodontitis. PPD of 6 mm or more, horizontal bone loss, also potential vertical bone loss, and may have furcation involvement of class II or III [7,12].

In the present study, the data did not include patients in stage IV; however, stage IV can be summarized as follows:

Stage IV: CAL 5 mm or greater, RBL reaches the mid-third or beyond, and five or more teeth have been lost due to periodontitis. PPD of 6 mm or more, horizontal bone loss, also potential vertical bone loss, may have furcation involvement of class II or III, and complex rehabilitation may be required due to factors such as masticatory dysfunction, secondary occlusal trauma or bite collapse (the patient may also have fewer than 20 teeth or fewer than 10 opposing pairs of teeth) [7,12].

### 2.5. Age, WWI and Other Predictors

The prevalence of many diseases increases with age, and this is also an essential feature of periodontitis [2,50,51,52,53]. Although studies show that the prevalence and severity of periodontitis increases as people get older, the underlying mechanisms influencing susceptibility are not fully understood [51,54,55,56]. Statistics explain the age-related increase in the total number of periodontitis patients by an increase in the prevalence of moderate periodontitis [2,51]. Furthermore, the prevalence of severe periodontitis can be conservatively estimated to be the same worldwide in all age groups over 45 years of age, with estimates ranging from 15% on either side [2,51,57]. Age was therefore a noteworthy predictor in the modeling.

In addition, age was examined as a factor in a variable that also included a waist-to-height ratio (WHtR). We hypothesized that waist-to-height ratio and age together could provide a variable that would effectively predict periodontitis. WHtR has been found to be associated with the number of deeper periodontal pockets (4 mm or more) [58]. Interestingly, waist circumference is also related to other predictive factors, such as the weight-adjusted waist index (WWI). It has been suggested that WWI is positively correlated with periodontitis, especially in cases of moderate-to-severe disease [59]. WWI was investigated as a predictor alone and in combination with other factors predicting the presence of periodontitis and its stages.

Diabetes was among the predictive variables studied in this research, not least due to the extensive research conducted on its association with periodontitis [1,3,60,61]. For example, in a large study in the United States (NHANES III), evidence supported an association between poorly controlled type 2 diabetes mellitus and severe periodontitis [62]. Epidemiological studies confirm that there is a two-way interlinked relationship between diabetes and periodontitis, with diabetes increasing the risk for periodontitis, and periodontal inflammation negatively affecting glycemic control [1]. Diabetes was an obvious candidate variable in the predictive model, and all patients participating in the study underwent an HbA1c blood test, where a result of ≥6.5% indicated that the patient had diabetes.

Tobacco smoking was also a predictor, just like diabetes, which was expected to be useful in modeling. Tobacco smoking is the most important risk factor for chronic destructive periodontal disease, and the severity of periodontitis is linked to the amount of smoking [63,64,65,66]. Smoking has a detrimental effect on incidence and progression of periodontitis [66].

### 2.6. Sample Data

The research material consisted of 149 Greek adult patients who visited Periodontology University Clinic and General Army Hospital in Thessaloniki, Greece. Informed consent was confirmed by the participants’ signatures, and the research was conducted in accordance with the protocol outlined by the Research Committee, Aristotle University of Thessaloniki, Greece, and approved by the Ethical Committee of the School of Dentistry (protocol number #64, 12 June 2018). All procedures performed in the present study involving human participants were in accordance with the ethical standards of the institutional and/or national research committee and with the 1964 Helsinki declaration and its later amendments or com-parable ethical standards [18].

Selection criteria for the study: age ≥ 18 years (both genders), number of teeth present ≥ 15 teeth, patient physical status 1 or 2 in A.S.A. classes according to the classification of American Society of Anesthesiology, the written informed consent of the research participants and the result of the self-assessed questionnaire proposed by the Centers for Disease Control and Prevention (CDC, Atlanta, GA, USA), for patients at high risk of developing Diabetes Mellitus ≥ 9. Exclusion criteria of the study: presence of diabetes mellitus or immunomodulatory diseases, a medication intake that affects glycemic control, periodontal therapy for the last six months and women in pregnancy or lactation [18].

During the 2017–2018 study period, a single calibrated examiner (A.G.) performed all clinical assessments on patients [18]. For each participant, key clinical measures of periodontitis and oral health status were recorded on six surfaces per tooth, excluding third molars. Also, anthropometric parameters and related factors such as weight, height, waist circumference, tobacco smoking status (yes/no), and age were recorded. (The waist circumference was measured at the point located midway between the top of the hip bone and the bottom of the ribs.) No missing data were reported for this cross-sectional observational study, which followed the guidelines of the STROBE statement. Table 1 presents the key information of the study.

### 2.7. Mathematical Background

In this study, predictive functions were generated using logistic regression analysis and variables were selected using a backward stepwise method [67,68,69,70,71,72,73]. Multicollinearity between variables was examined using the variance inflation factor (VIF) and autocorrelation was checked using scatterplots. Standardized residuals exceeding ±3.0 were used to indicate that the regression results should be investigated more closely for outliers, for example. It was also agreed that in the case of these anomalies, leverage values would be scrutinized to determine how extreme these particular data points would be relative to other data points. A Cook’s distance of 0.5 was another threshold set for examining potential sample deviations and assessing how much the estimates of the regression model would change when a particular data point was omitted from the analysis.

The publications of Peduzzi et al. [74] and Long [75] were used to assess whether the sample size was sufficient for modeling polynomial functions using logistic regression analysis. Peduzzi et al. suggest that the number of events per variable (EPV) ratio of less than 10 may lead to major problems [74]. (The number of events refers to the lower total number of possible outcomes, i.e., if, for example, there are 50 healthy and 100 periodontitis patients in the patient sample data, the group of healthy is the determining factor.) A criterion was set that the EPV should be at least 10. Additionally, in line with Long’s caution against using multiple logistic regression with a sample size below 100, this value was established as the minimum sample size [75]. In the case of simple logistic regression, i.e., logistic regression performed using a single predictor, we also examined the results carefully, although the absolute limit of 100 samples was associated with multiple logistic regressions.

**Figure 2:**
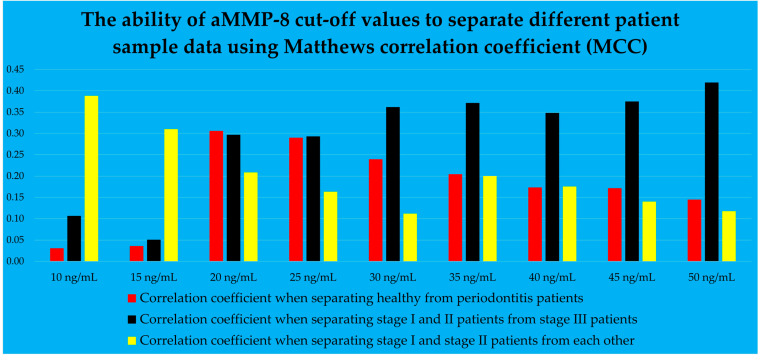
The mouth rinse aMMP-8 cut-off values, described by Matthews correlation coefficient.

The following statistical metrics were considered when examining the results of the study: sensitivity, specificity, accuracy, chi-square goodness-of-fit test, phi coefficient φ for categorical data, and area under the receiver operating characteristic (ROC) curve (AUC). Additionally, the F1 score was calculated to assess the performance of the generated functions, for example alongside accuracy, and the Matthews Correlation Coefficient (MCC) was employed to interpret the results presented in the bar diagram in Figure 2 of this study [40]. Youden’s index was applied in determining the optimal threshold for logistic regression functions, and Wilson score with continuity correction was utilized to calculate confidence intervals for sensitivity, specificity, and stage prediction accuracy, including stage-specific accuracies [76,77]. It was agreed that the significance level *p* for all predictor variables of the functions developed using logistic regression analysis was less than 0.05. The significance level of the chi-square goodness-of-fit test was also <0.05. The logistic regression and parameters directly related to it, for example, std. residuals and Cook’s distances, and the calculations of AUC were conducted using IBM SPSS Statistics (version 31) software. All other calculations, including the results of the chi-squared goodness-of-fit test and Wilson scores with continuity correction presented in the study, were performed manually.

## 3. Results

Three predictive functions were created to form a model that determines the presence and stage of periodontitis. The first function was used to distinguish healthy patients from those with periodontitis. After revealing periodontitis patients, a second function was used to differentiate stage I and II patients from stage III patients. The third function separated stage I and II patients from each other. In order to model the above functions, the optimal aMMP-8 cut-off values, in ng/mL, were obtained from the results of the Oralyzer digital reader. The study also examined the potential use of aMMP-8 concentration as a continuous predictive variable. Figure 2 graphically shows the ability of aMMP-8 cut-off values to discriminate different patient sample data using the Matthews correlation coefficient (MCC) for binary data, and values of 10, 15, 20, 25, 30, 35, 40, 45 and 50 ng/mL were investigated [40].

From Figure 2, it can be observed that the red bars represent the correlation coefficients used to determine which aMMP-8 concentration was employed to separate healthy patients from those in stages I, II, and III. The red bars peaked at 20 ng/mL. This cut-off was used as the primary selection for the aMMP-8 concentration as a predictor in the model to distinguish healthy patients from periodontitis patients. Furthermore, in Figure 2, the black bars on the right side of the graph showed higher correlation coefficients compared to the black bars on the left side of the table. A cut-off of 50 ng/mL was used as the first-choice aMMP-8 concentration cut-off for modeling the differentiation of stage III patients from stage I and II patients.

The yellow bars in Figure 2 illustrate how aMMP-8 cut-off values can be used to separate stage I and stage II patients. The bar graph indicates that the cut-off values of 10 and 15 ng/mL depict the highest bars, and these were examined as the main predictive factors in this scenario. The change in the heights of the yellow bars is opposite compared to the bars colored black. This seems logical because the yellow bars represent a group of patients which have less severe periodontitis than the patients in the case of black colored bars. The stage of periodontitis is associated with the concentration of aMMP-8 in mouth rinse [17].

The mouth rinse aMMP-8 cut-off value, which represented the highest correlation coefficient, was not automatically selected as the optimal cut-off value for detecting periodontitis or its stage in the given function question cases. Instead, the cut-off value was chosen that would most effectively match the other predictor variables and at the same time give the most accurate results. Additionally, during the modeling process, aMMP-8 concentration in mouth rinse was also tested as a continuous value and the results were reviewed to ensure that the most accurate prediction results were achieved.

The following aMMP-8 levels were used in the modeling: healthy individuals were separated from stages I, II and III by a variable including cut-off of 20 ng/mL as a factor, aMMP-8 cut-off of 50 ng/mL was used to separate stage III from other periodontitis patients, and in the modeling scenario of separating stage I and II patients from each other, a continuous value of aMMP-8 concentration was selected as a predictor with two other explanatory factors. This special variable included a total of three components: aMMP-8 concentration as a continuous number, as well as VPI and the number of teeth present.

**Figure 3:**
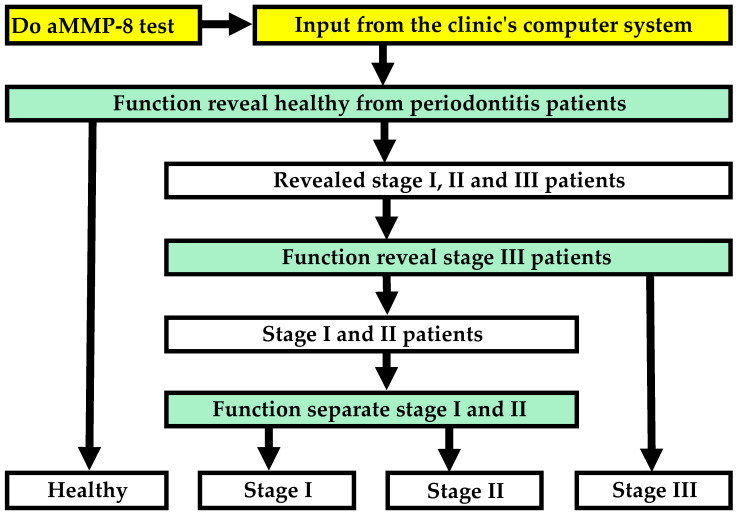
Flowchart illustrating how the model detects periodontitis and its stages.

**Figure 4:**
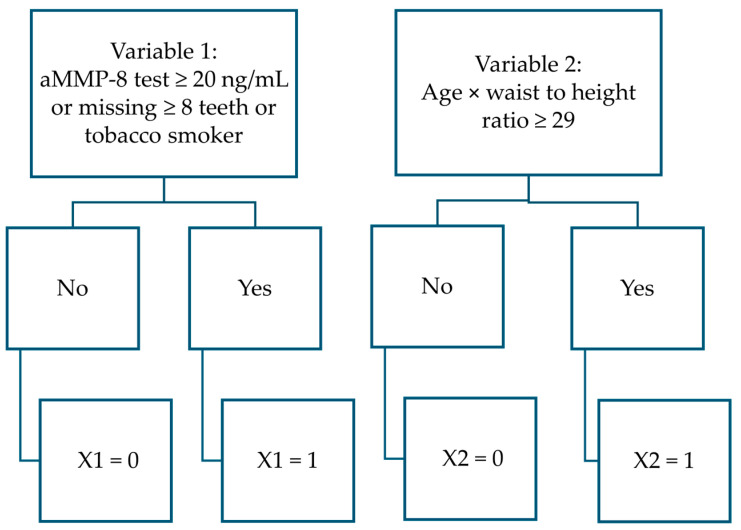
Flowchart to determine variable values in PERIORISK function.

**Figure 5:**
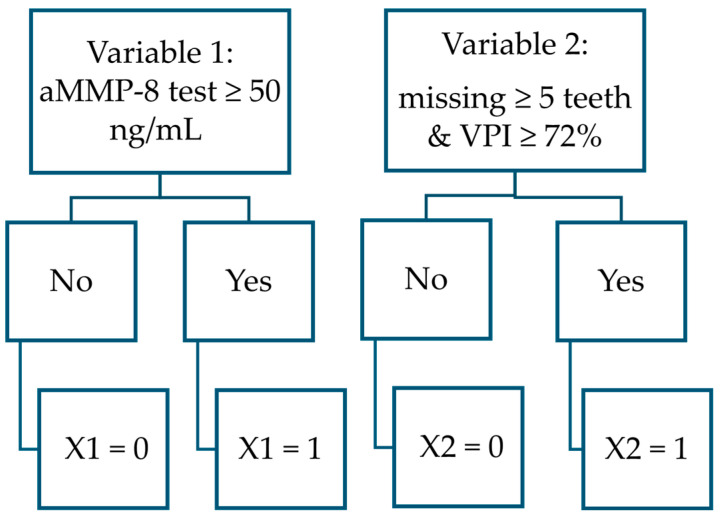
Flowchart to determine variable values in PERIOSTAGE III function.

**Figure 6:**
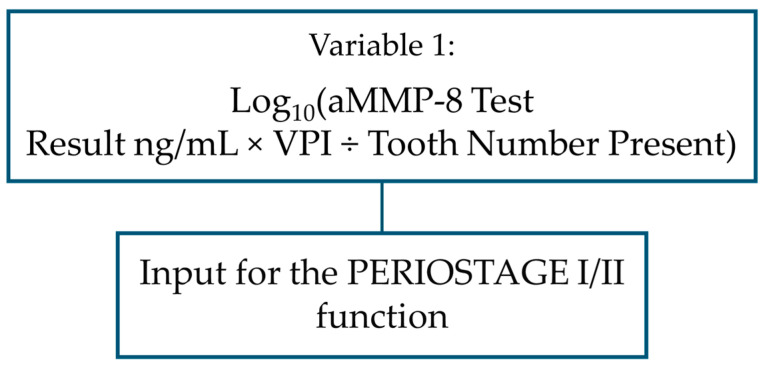
Flowchart illustrating the use of the PERIOSTAGE I/II function.

Altogether, eleven explanatory factors were selected to create the five variables for the three different predictive polynomial functions to form the complete model of this study (Table 2, Figure 3, Figure 4, Figure 5 and Figure 6):

- aMMP-8 mouth rinse test result (cut-off value of 20 ng/mL)
- aMMP-8 mouth rinse test result (cut-off value of 50 ng/mL)
- aMMP-8 mouth rinse test result, examined as a continuous value at 5-point intervals in ng/mL
- Tobacco smoking status
- The visible plaque index (VPI) measured as a continuous value with a precision of two decimal places
- Visible plaque index VPI ≥ 72%
- Number of teeth present
- Number of teeth present: ≥5 teeth missing
- Number of teeth present: ≥8 teeth missing
- Age
- Waist-to-height ratio

The polynomial function developed in this study for distinguishing healthy patients from those with periodontitis:

$$PERIORISK\;=\;\frac{1}{1+e^{-\left(3.399\;\times \;X1\;+\;2.998\;\times \;X2\;-\;1.259\right)}}$$

In which,

PERIOSRISK = result predicting whether the patient has periodontitis [No.] (If the result ≥ 0.536, the patient statistically has periodontitis; otherwise = the patient is not statistically considered to have periodontitis).

X1 = aMMP-8 test ≥ 20 ng/mL or a tobacco smoker or missing ≥8 teeth [No.] (yes = 1, no = 0; patient boundary conditions are: aMMP-8 concentration in mouth rinse ≥ 5 ng/mL and ≤85 ng/mL, 15 ≤ teeth number present ≤ 28).

X2 = age × waist to height ratio ≥ 29 (with two decimal places, i.e., 28.99 < 29) [No.] (yes = 1, no = 0; patient boundary conditions are: 25 yrs. ≤ age ≤ 78 yrs., WC 60 cm–152 cm, height 150–193 cm, waist to height ratio 13.50–56.42).

The polynomial function developed in this study for distinguishing stage III patients from stage I and II patients:

$$PERIOSTAGE\;III\;=\;\frac{1}{1+e^{-\left(2.328\times X1+3.252\times X2-2.216\right)}}$$

In which,

PERIOSTAGE III = result predicting whether the patient has stage III or stage I/II [No.] (If the result ≥ 0.313, the patient statistically has stage III periodontitis; otherwise, the patient is statistically considered to have stage I/II periodontitis).

X1 = aMMP-8 test ≥ 50 ng/mL [No.] (yes = 1, otherwise = 0; patient boundary conditions are: aMMP-8 concentration in mouth rinse ≥ 5 ng/mL and ≤85 ng/mL).

X2 = Missing ≥ 5 teeth and VPI ≥ 72% [No.] (yes = 1, otherwise = 0; wisdom teeth are excluded from the count; patient boundary conditions are: 15 ≤ teeth number present ≤ 28, 0.64% ≤ VPI ≤ 100.00%).

The polynomial function developed in this study for distinguishing stage I and stage II patients from each other:

$$PERIOSTAGE\;I/II\;=\;\frac{1}{1+e^{-\left(1.408\times X1-0.035\right)}}$$

In which:

PERIOSTAGE I/II = result predicting whether the patient has stage I or stage II [No.] (If the result ≥ 0.816, the patient statistically has stage II periodontitis; otherwise, the patient is statistically considered to have stage I periodontitis).

X1 = log_10_(aMMP-8 test result in mouth rinse × VPI ÷ number of teeth present [No.] (wisdom teeth are excluded from the count; patient boundary conditions are: aMMP-8 concentration in mouth rinse ≥ 5 ng/mL and ≤60 ng/mL, 0.64% ≤ VPI ≤ 100.00%, 16 ≤ teeth number present ≤ 28).

Based on the results of the regression analyses conducted in this study, it was found that the sensitivity, specificity, chi-square goodness-of-fit test, phi coefficient φ for categorical data, and area under the ROC curve (AUC) of the developed functions reached the acceptance limit. The accuracy results, combined with the F1 scores, were also considered to be at a sufficient level.

The PERIORISK function demonstrated a sensitivity of 94%, a specificity of 74%, and an accuracy of 90%. The PERIOSTAGE I/II function, in turn, showed a sensitivity of 83%, a specificity of 57%, and an accuracy of 79%. The PERIOSTAGE III function yielded a sensitivity, specificity, and accuracy of 57%, 95%, and 87%, respectively. All the functions were tested on the same sample data from which they were generated.

- PERIORISK: sensitivity 111/118 = 0.94, specificity 23/31 = 0.74; chi-square goodness-of-fit test χ^2^ (1, *N* = 149) = 0.04, *p* = 0.838; phi coefficient φ = 0.69; AUC = 0.915 (95% CI = 0.865–0.964); Youden’s index = 0.683, cut-off 0.536; accuracy 134/149 = 0.90, F1 score 0.94.
- PERIOSTAGE I/II: sensitivity 67/81 = 0.83, specificity 8/14 = 0.57; chi-square goodness-of-fit test χ^2^ (1, *N* = 95) = 3.79, *p* = 0.052; phi coefficient φ = 0.33; AUC = 0.779 (95% CI = 0.663–0.894); Youden’s index = 0.399, cut-off 0.816 (The highest Youden’s index to determine the performance of the function, i.e., 0.610, yielded a cut-off value of 0.857, was not used in the model in this study because the selected Youden’s index of 0.399 (cut-off value 0.816) produced 75 correct stage predictions, while the aforementioned highest index resulted in two fewer, i.e., 73 correct stage predictions [76]); accuracy 75/95 = 0.79, F1 score 0.87.
- PERIOSTAGE III: sensitivity 13/23 = 0.57, specificity 90/95 = 0.95; chi-square goodness-of-fit test χ^2^ (1, *N* = 118) = 1.64, *p* = 0.201; phi coefficient φ = 0.56; AUC = 0.764 (95% CI = 0.633–0.895); Youden’s index = 0.513, cut-off 0.313; accuracy 103/118 = 0.87, F1 score 0.63.

In the PERIORISK function, the number of events per variable (EPV) was found to be 15.5, as two variables were used and 31 patients were in the group, which represented a smaller proportion of potential outcomes (31/2 yields an EPV of 15.5). The PERIOSTAGE III function included two variables, and this results in an EPV of 23/2, which equals 11.5. The EPV of the PERIOSTAGE I/II function was 14 because the sample data consisted of 14 patients and only one variable was used in the function. Therefore, adhering to the suggestion by Peduzzi et al. for a minimum of 10 EPV, this criterion was met by all three functions presented in the study [74]. Furthermore, in accordance with Long’s recommendation that multiple logistic regression should not be performed if the sample size is less than 100, this criterion was also met in the development of all functions. (the PERIORISK function was created using data from 149 patients, and in the PERIOSTAGE III function, the sample size was 118 patients) [75]. Given these aforementioned results, the sample sizes for the logistic regression analyses and their derived polynomial functions were deemed sufficient, as they fulfilled the sample size criteria established by Peduzzi et al. and Long [74,75]. The PERIOSTAGE I/II function, which was based on a dataset of 95 samples, was modeled using a single variable; consequently, the 95 samples were considered sufficient for its development.

Of the three functions created in this study, only PERIOSTAGE I/II possessed a continuous predictor variable, and the assumption of linearity was examined from scatterplots. The predicted log-odds demonstrated a linear relationship with the continuous explanatory variable. And thus, the linearity assumption was met for the PERIOSTAGE I/II function. Multicollinearity was checked with VIF for functions with two variables, and analysis confirmed that there was no problematic correlation among the independent variables. (The VIF value was found to be 1.047 for PERIORISK function and for the PERIOSTAGE III VIF value yielded 1.163.) Also, the residuals were plotted against the chronological patient order, and no autocorrelation was observed in any of the three functions.

For outliers, one patient sample in the PERIOSTAGE I/II function data exceeded the ±3.0 threshold for standardized residuals (i.e., −3.2). The patient was predicted to be in the stage II sample group but was diagnosed with stage I [7,47,48]. When the regression analysis was conducted without the potential outlier, the results of the statistical analyses, such as sensitivity and specificity, were almost the same. The results only changed for this one incorrect prediction, as it was no longer present in the outcomes. Cook’s distance of the sample was 0.1; no samples in this study had a Cook’s distance > 0.5. The leverage of the aforementioned potential outlier was found to be 0.014, which was below the average of all leverage values in this 95-sample dataset (i.e., 0.021). After careful consideration, it was decided that this data point had a relatively low probability of unduly influencing the model, and it was concluded that there were insufficient grounds to remove the data point as an outlier.

The complete model detected patients without periodontitis in 74.2% of cases (95% CI: 55.1–87.5%, sp. 23/31) and patients with periodontitis in 94.1% of cases (95% CI: 87.7–97.4%, se. 111/118), and in these the correct stage was detected in 71.2% of cases (95% CI: 61.7–79.2%, 79/111 = 0.712). Stage-specific accuracies: stage I 57.1% (95% CI: 29.7–81.2%), stage II 78.4% (95% CI: 67.0–86.8%), and stage III 56.5% (95% CI: 34.9–76.1%). (Stage I: 8/14 = 0.571; stage II = 58/74 = 0.784; stage III 13/23 = 0.565.) Wilson score with continuity correction was applied to calculate the confidence intervals presented in this section [77].

The complete model was tested on the same patient data from which it was originally created. The performance calculations, which met all required acceptance criteria, supported the conclusion that the complete model demonstrated sufficient performance in this study.

## 4. Discussion

This paper proposes a framework for developing a statistical model for use in dental clinics to facilitate the diagnosis of periodontitis and its stages. The method uses an aMMP-8 mouth rinse point-of-care test, the value of which is read from a digital reader in five minutes. In general, the data needed to use the predictive model is in the computer system of the dental clinic and can be utilized in real-time, so the dental professional has all the statistical benefits in their work.

The model successfully differentiated between healthy individuals and periodontitis patients, and could also classify the stage of periodontitis, with promising predictive accuracy. The findings of this study supported the hypothesis that a predictive model for periodontitis and its stages could be developed using five to twenty factors from dental clinic data. Patients without periodontitis were identified in 74% of cases and patients with periodontitis were revealed with a success rate of 94% and of these, the correct stage was determined in 71% of cases. Compared to a previously published similar prediction model, the rate of correct prediction of periodontitis stage increased from 61% to 71% [20]. Although this increase was calculated by comparing the model of this study to a rapid test for home-use, it can be concluded that improvements of 10% are noteworthy. The double-digit percentage increase was due to the use of a digital reader, which included measurements of mouth rinse aMMP-8 concentration on a continuous scale expressed in ng/mL.

The introduction of statistical prediction methods offers the opportunity to advance the diagnostic process of periodontitis. Since early diagnosis of periodontitis has been identified as one of the most important areas for improvement in periodontitis diagnostics, this study provides a framework for improving the discrimination of stage I patients in particular [7,8]. Furthermore, as the data of this study did not include stage IV patients and problems have been reported in distinguishing stage III and IV patients, the basic principle of this study is also applicable to solving these problems [8]. The prevalence of “grey areas” in diagnosis creates a need for additional training, and the results of this study could help by directly producing statistics for diagnostic aid or by providing research for educational materials [8,11,12].

The modeling of the study data began by examining the most easily observable variable, which was the patient’s age. This predictor, which is available from the dental clinic’s computer system, was found to guide the modeling in a way that, in the first step, healthy and periodontitis patients were distinguished. The modeling process included a clear understanding that age is a very reliable and easy-to-use statistical predictor of periodontitis. It is well-known that the prevalence of periodontitis increases with age and in the United States, for instance, it has been reported that 68 percent of adults aged over 65 with teeth have periodontitis [78,79]. As a predictor variable, age was combined with information of patient’s waist-to-height ratio (WHtR). This customized variable, age × WHtR, provided a variable that effectively predicted periodontitis. WHtR has been found to be associated with the number of teeth with deeper (4 mm or more) periodontal pockets [58]. Next, factors related to aMMP-8 concentration in mouth rinse (cut-off value 20 ng/mL) and tobacco smoking were added to the prediction function, and the modeling results reached an acceptably high level [17,63]. Finally, the number of teeth present was also added to the prediction function as an explanatory factor. Information on the number of teeth lost due to periodontitis is part of the classification of periodontitis, and periodontitis is the most common cause of tooth loss [7,47,48].

Since the prediction model uses the aMMP-8 digital reader results, the modeling was not limited to using a single mouth rinse concentration value, such as 20 ng/mL, which is a practical approach for home testing as a personalized medicine approach [19,20,21]. Figure 2 illustrates the vast possibilities of this study to use various aMMP-8 cut-off values in mouth rinse to discriminate patients across different sample data scenarios. It has been observed that the levels of aMMP-8 in mouth rinse are positively associated with the stage of periodontitis, and Sorsa et al. have proposed their inclusion in the new periodontal disease classification system since 2020 [17].

After separating healthy patients, the model differentiated stage III patients from stage I and II patients. It is evident again, from Figure 2, from the patient separation scenario of stage III patients (black colored bars), that the higher the aMMP-8 test result in mouth rinse (ng/mL), the more likely the patient is to have stage III of periodontitis, as measured by MCC [40]. This is logical, as ongoing active periodontal tissue damage (i.e., active phase of periodontitis) can be identified in oral fluids as an increase in aMMP-8 levels [17,80,81]. Furthermore, the more active the disease is, the more likely it is that the patient will progress to the next stage of periodontitis. The model presented in this study is entirely statistical, as the previous chain of reasoning shows.

In the previously discussed stage III patient separation scenario, the visible plaque index (VPI) was utilized in the predictive model. In fact, VPI fit both stage prediction functions presented in this study, however, always as one explanatory factor among other factors. There seems to be no debate about the suitability of dental plaque for predicting periodontitis per se, but its impact on determining the stage in different modeling scenarios should be investigated using comprehensive data [17,20,82,83]. This may provide future research topics in which VPI, or other dental plaque indices have an essential impact on determining the stage of periodontitis. This is interesting if only because it has been proposed since the 1990s that dental plaque has the potential to serve as a reservoir and a site of, for example, MMP-8 activation in periodontal inflammation (i.e., the enzyme in the center of this article) [84].

In the third and final part of the prediction model, stage I patients were separated from stage II patients. However, since there were 14 stage I patients in the dataset, this allowed for the use of only one predictor variable in the PERIOSTAGE I/II function. (The sample size requirements for logistic regression in this study included the criterion that there should be at least 10 patients for each variable from the smaller part of the binary sample [74]). The most precise variable for modeling the differentiation of stage I and II patients was found to be a predictor with three factors: aMMP-8 concentration as a continuous number, VPI, and number of teeth present. The first two factors were multiplied with each other and then divided by the third factor, after which the logarithm of the result was taken. (The logarithm of the predictor was implemented to improve the model’s fit regarding the residuals. The performance of the generated prediction function remained unchanged in this particular case regardless of whether the logarithm of the variable was taken or not, and was the same in terms of, for example, sensitivity, specificity, etc.). The variable allowed for the differentiation of stage I and stage II patients with sufficient accuracy. It is worth mentioning that if the sample data had included more stage I patients, it is likely that more patients with aMMP-8 cut-offs below 10 or 15 ng/mL would have been in the dataset [17]. In this case, with a larger dataset, these cut-offs could have been used as predictor factors in the PERIOSTAGE I/II function created (yellow bars on the left side of Figure 2). Furthermore, it can be predicted that lower aMMP-8 concentration results (<20 ng/mL) may play a key role in the future, when stage I patients are detected with greater accuracy in future prediction models.

The study investigated bleeding on probing (BOP) as a predictive variable, particularly in the assessment of the stages of periodontitis. BOP was found to be a promising factor in distinguishing between patients with stage I and stage II. However, in the sample data of this study, BOP could not be included in the predictive model because other explanatory factors, such as mouth rinse aMMP-8 concentration, VPI, and number of teeth present, were more suitable for modeling. It is worth mentioning that since the number of variables can be increased with larger sample sizes, BOP is at least a promising predictor according to the results of this study, and its investigation may prove to be worthwhile. Weight-adjusted waist index (WWI) was also a welcome new variable candidate in the model and showed potential in differentiating stage III patients from other periodontitis patients [59]. However, for the same reason as BOP, the number of variables was a limiting factor and WWI was not selected as a predictor variable in this study.

Diabetes, which is a well-studied predictor of periodontitis and its stages, was an apparent variable that warranted attention in this study [1,3,60,61,62]. Diabetes has been an important predictor in previously published models similar to this study [19,20]. The same observation was made in this model created for dental clinics; however, as previously mentioned in this study, diabetes was also one of those predictors that could not be utilized due to the availability of so many other candidates.

The results of the study showed that the model identified stage II patients with a high success rate of 78%. The model achieved 57% accuracy in detecting both stage I and stage III patients. Including more stage I and III patients in the sample, which allows the number of predictive variables to be increased, the success rate of stage detecting is expected to improve substantially.

The next step in developing the presented model is to examine all of the aforementioned predictive factors using larger and more comprehensive datasets. Tobacco smoking is a fine example of how, given all the opportunities offered by data diversification, and the already promising research results, the accuracy of the model can be predicted to increase in the future. The amount of smoking and history of smoking should be taken into account in future versions of the models, as these factors were not available in the data of this study [63,64,65,66].

Additionally, research topics such as personal oral hygiene, medication use, blood pressure, or gender are interesting subjects that can bring significant refinements to the model [85,86,87,88]. Intriguing fields of periodontitis research include also genetic factors. At present, variants of at least 65 genes have been suggested to be associated with periodontitis. However, the identification of genetic factors associated with or actually contributing to the pathogenesis of periodontitis has been limited, and validation results have been modest [89].

It is worth mentioning that the models produced by the presented method are not the same in different populations. The model is completely statistical and predicts periodontitis and its stages using statistical parameters from the sample data from which it is derived. It is important to emphasize that the order of patient data separation may be different between models and some predictive models may require more than three predictive functions. The cut-off values for aMMP-8 concentrations used in the functions may vary. The details of the sample data will certainly produce different coefficients for predictive functions, but the main idea should be essentially the same. Both the aMMP-8 concentration in mouth rinse and the dental plaque on the tooth surface, as well as the number of teeth present, have consistently been associated with periodontitis and its stages in different populations [7,17,46,84,90,91]. Tobacco smoking and age are also directly related to periodontitis and its stages [2,51,52,53,63,64,65,66,92,93]. It can therefore be concluded that the variables should be suitable and applicable to different populations.

The proposed method in this study needs to be validated in future studies to ensure its generalizability and robustness. The validation should be conducted with larger datasets even though the chi-square goodness-of-fit test, for all three created predictive functions, concluded that there was insufficient evidence to suggest that the observed data significantly deviated from the expected distribution (the null hypothesis, i.e., the observed data does follow the expected distribution, was not rejected; none of the chi-square goodness-of-fit test *p*-values were <0.05). In validating the models of the introduced method, cross-validation is a useful approach to confirm the results. There are many variations of cross-validation, but the idea is to test the model’s ability to predict new data that was not used in creating the model (i.e., using one part of the sample data for modeling and the rest of the data for testing) [94,95]. Bootstrapping is another interesting way to verify the results if the data is considered, e.g., limited. In bootstrapping, the original sample data is used to create multiple bootstrap samples from which statistical estimates are obtained to describe the original data [95]. (Bootstrap sample can be the same size as the original dataset and essential in bootstrapping is that each time a “random single sample” is taken from the original data in the formation of the bootstrap sample, it is returned to the original data before the next “random single sample” is selected).

In general, more extensive studies are needed to validate accuracy in all stages of periodontitis, and reliability also requires more thorough investigation. The models of the presented method should achieve reliability, where the predicted results would be repeated in as many cases as possible. Intra-rater measurements are essential, for instance, to ensure that the predictions of the patients’ periodontitis status are consistent [96]. To ensure inter-rater reliability, at least two users of the model should be involved in the test, and the results produced by the model should achieve sufficiently similar results for all users. Inter-rater measurements are suitable, for example, when several people participate in data collection [96,97]. In addition, regulation of the presented method is important to ensure that all legal and privacy issues are properly addressed [98].

Looking a little further into the future, the development of the method presented could potentially reduce radiation exposure at the population level if it would allow the determination of the presence and stages of periodontitis without X-rays in certain cases. It could also help optimize dental resources by improving timely patient care at different stages of periodontitis. In addition, statistical issues related to periodontitis could possibly be explored more broadly, as the framework presented in this study could facilitate the storage of large research databases in a way that would help in the development of new innovations or variations on current methods. Finally, it would be more than desirable if the results of this study could, in their own way, accelerate the development of disease detection related to periodontitis and its stages [1,3,4,5,6,99].

## 5. Conclusions

According to our information, this is the first time a framework has been proposed for the development of a model that utilizes the results of mouth rinse aMMP-8 levels from a digital reader to determine the presence and stages of periodontitis. The model variables can be used directly from the computer systems of dental clinics, supporting real-time diagnosis in a way that provides dental professionals with all the statistical advantages in their work. The method is entirely statistical and is intended for dentists, dental hygienists and dental nurses. The modeling results for determining periodontitis and its stages were found to be sufficient and promising. We propose wider implementation of this method across different populations for professional use in dental clinics.

## Figures and Tables

**Table 1:** General characteristics of the patient data. For continuous variables, data are shown as mean ± SD. For categorical variables, data are shown as a count. WWI, weight-adjusted waist index; aMMP-8, active-matrix metalloproteinase-8; CAL, clinical attachment level; PPD, periodontal probing depth; VPI, Visible Plaque Index; BOP, bleeding on probing.

Patient’s Data	Stage of Periodontitis Status			
	No Evidence of Periodontitis (*n* = 31)	Stage I (*n* = 14)	Stage II (*n* = 81)	Stage III (*n* = 23)
**Gender**				
Female	11	13	39	12
Male	20	1	42	11
**Smoking status**				
Tobacco smoker	3	7	24	10
Non-smoker	28	7	57	13
**Diabetic status**				
Diabetic	0	0	3	4
Non-diabetic	31	14	78	19
**Age (years)**	43 ± 11	62 ± 8	55 ± 10	56 ± 10
**Body mass index (kg/m^2^)**	30.6 ± 4.5	28.6 ± 4.4	30.5 ± 4.7	29.3 ± 5.9
**Weight (kg)**	93 ± 17	78 ± 11	89 ± 17	85 ± 23
**Height (cm)**	174 ± 10	165 ± 6	171 ± 9	169 ± 10
**Waist circumference (cm)**	100 ± 17	98 ± 12	103 ± 14	105 ± 21
**Waist-to-height ratio (cm/cm)**	0.57 ± 0.08	0.60 ± 0.09	0.60 ± 0.08	0.62 ± 0.11
**WWI (cm/√kg)**	10.36 ± 1.02	11.16 ± 1.08	10.93 ± 1.01	11.49 ± 1.23
**aMMP-8 levels**				
aMMP-8 ≥ 20 ng/mL	2	2	31	17
aMMP-8 < 20 ng/mL	29	12	50	6
aMMP-8 ≥ 50 ng/mL	0	0	3	8
aMMP-8 < 50 ng/mL	31	14	78	15
aMMP-8 (ng/mL)	15 ± 5	15 ± 10	20 ± 10	40 ± 25
**Stage of periodontitis status**				
No evidence of periodontitis	31	0	0	0
Stage I	0	14	0	0
Stage Il	0	0	81	0
Stage III	0	0	0	23
**Grade of periodontitis status**				
No evidence of progression	31	0	0	0
Grade A	0	7	7	0
Grade B	0	7	70	13
Grade C	0	0	4	10
**CAL (mm)**	2.4 ± 0.5	2.3 ± 0.5	3.4 ± 0.8	4.8 ± 1.2
**PPD (mm)**	2.2 ± 0.3	2.2 ± 0.4	3.0 ± 0.7	3.9 ± 0.9
**Number of teeth present (No.)**	27 ± 2	25 ± 2	24 ± 3	22 ± 4
**VPI (%)**	43 ± 22	29 ± 20	48 ± 27	63 ± 28
**BOP (%)**	42 ± 25	33 ± 17	56 ± 23	63 ± 22

**Table 2:** Coefficient output data of multiple logistic regression modeling of the three functions created.

Functions and Variables	B	S.E.	Wald	df	Sig	Exp(B)
**PERIORISK: function separating healthy patients from stage I, II and III patients**						
aMMP-8 test ≥ 20 ng/mL or tobacco smoker or missing ≥8 teeth	3.399	0.716	22.554	1	<0.001	29.937
Age × waist-to-height ratio ≥ 29 yrs. × cm/cm	2.998	0.626	22.941	1	<0.001	20.054
Constant	−1.259	0.428	8.658	1	0.003	0.284
**PERIOSTAGE III: function separating stage III patients from stage I and II patients**						
aMMP-8 test ≥ 50 ng/mL	2.328	0.838	7.714	1	0.005	10.253
Missing ≥ 5 teeth and VPI ≥ 72%	3.252	0.871	13.960	1	<0.001	25.853
Constant	−2.216	0.333	44.182	1	<0.001	0.109
**PERIOSTAGE I/II: function separating stage I and stage II patients from each other**						
log_10_ (aMMP-8 concentration in mouth rinse in ng/mL × VPI ÷ number of teeth present)	1.408	0.564	6.238	1	0.013	4.089
Constant	−0.035	0.728	0.002	1	0.962	0.966

## Data Availability

The data presented in this study are available on request from the corresponding author due to privacy and legal reasons.
